# Evaluation of the effects of botulinum toxin A injections when used to improve ease of care and comfort in children with cerebral palsy whom are non-ambulant: a double blind randomized controlled trial

**DOI:** 10.1186/1471-2431-12-120

**Published:** 2012-08-09

**Authors:** Megan Thorley, Samantha Donaghey, Priya Edwards, Lisa Copeland, Megan Kentish, Kim McLennan, Jayne Lindsley, Laura Gascoigne-Pees, Leanne Sakzewski, Roslyn N Boyd

**Affiliations:** 1 Queensland Cerebral Palsy Health Service, Royal Children Hospital, Brisbane, Australia; 2 Queensland Cerebral Palsy and Rehabilitation Research Centre, The School of Medicine, The University of Queensland, Brisbane, Australia

**Keywords:** Botulinum Toxin A, Cerebral Palsy, Pain, Comfort, Double blind randomized controlled trial

## Abstract

**Background:**

Children with cerebral palsy (CP) whom are non-ambulant are at risk of reduced quality of life and poor health status. Severe spasticity leads to discomfort and pain. Carer burden for families is significant. This study aims to determine whether intramuscular injections of botulinum toxin A (BoNT-A) combined with a regime of standard therapy has a positive effect on care and comfort for children with CP whom are non-ambulant (GMFCS IV/V), compared with standard therapy alone (cycle I), and whether repeated injections with the same regime of adjunctive therapy results in greater benefits compared with a single injecting episode (cycle II). The regime of therapy will include serial casting, splinting and/or provision of orthoses, as indicated, combined with four sessions of goal directed occupational therapy or physiotherapy.

**Method/design:**

This study is a double blind randomized controlled trial. Forty participants will be recruited. In cycle I, participants will be randomized to either a treatment group who will receive BoNT-A injections into selected upper and/or lower limb muscles, or a control group who will undergo sham injections. Both groups will receive occupational therapy and /or physiotherapy following injections. Groups will be assessed at baseline then compared at 4 and 16 weeks following injections or sham control. Parents, treating clinicians and assessors will be masked to group allocation. In cycle II, all participants will undergo intramuscular BoNT-A injections to selected upper and/or lower limb muscles, followed by therapy.

The primary outcome measure will be change in parent ratings in identified areas of concern for their child’s care and comfort, using the Canadian Occupational Performance Measure (COPM). Secondary measures will include the Care and Comfort Hypertonicity Scale (ease of care), the Cerebral Palsy Quality of Life Questionnaire (CP QoL–Child) (quality of life), the Caregiver Priorities and Child Health Index of Life with Disabilities Questionnaire (CPCHILD©) (health status) and the Paediatric Pain Profile (PPP) (pain). Adverse events will be carefully monitored by a clinician masked to group allocation.

**Discussion:**

This paper outlines the theoretical basis, study hypotheses and outcome measures for a trial of BoNT-A injections and therapy for children with non-ambulant CP.

**Trial registration:**

Australia New Zealand Clinical Trials Registry:N12609000360213

## Background

Cerebral palsy (CP) is “a group of permanent disorders of the development of movement and posture, causing activity limitation, that are attributed to non-progressive disturbances that occurred in the developing fetal or infant brain”. [1] p.9 Classification systems have been developed to indicate the severity of functional limitations in CP. The Gross Motor Function Classification System (GMFCS), developed by Palisano and colleagues in 1997, has become internationally accepted for the classification of gross motor abilities for children with CP [2]. The GMFCS is comprised of 5 levels, with GMFCS I reflecting the highest level of gross motor function. Children who are classified GMFCS IV and V are the most functionally impaired. Children classified as GMFCS IV require supportive seating for trunk control and to maximize upper limb function, and assistance for transfers. Self-mobility is limited to possible use of a powered wheelchair. Children who are classified as GMFCS V are unable to sit without support and have difficulty maintaining antigravity head and neck control. Children classified as GMFCS IV and V comprise approximately one third of children with CP in Australia [3].

The most common motor type of CP is the spastic type [3]. Spasticity is “a velocity-dependent resistance of a muscle to stretch”. [4] p.91 Spasticity commonly leads to muscle contractures and eventual bony deformities, which may result in decreased functional ability as a child’s development progresses [5,6]. Children whose CP is classified GMFCS IV or V frequently suffer from pain [7-9]. Pain is often associated with marked spasticity, spasms, skin breakdown, or postural positioning leading to an increased burden of care. Even simple tasks like dressing can become problematic when children have high levels of spasticity [10]. Pain negatively impacts children’s quality of life [9] and participation in schooling and family activities [8]. Pain is associated with a higher risk of stress for parents of children with CP [11]. Pain, discomfort and carer burden lead parents to seek treatments to reduce their child’s spasticity.

Spasticity can be reduced with intramuscular injections of botulinum toxin A (BoNT-A) [12]. When injected into a target muscle, BoNT-A enters the presynaptic terminal, and prevents the exocytosis of acetylcholine, thus reducing spasticity. The clinical effects have been reported to last for approximately 3-6 months. Intramuscular injections of BoNT-A, using Botox^®^ (Allergan PLC) have been approved (March 2011) by the Therapeutic Goods Association Australia for treatment of focal spasticity in the upper and lower limbs, including dynamic equinus foot deformity, in children with juvenile CP from the age of two years. Dysport^®^ is an alternative preparation of BoNT-A which will not be used in this study. Research has demonstrated the ability of BoNT-A to improve function for children with CP whom are ambulant [13-18]. When used with ambulatory children, BoNT-A is considered to be generally safe with relatively few and minor complications [19].

There is limited evidence for using BoNT-A with children with CP whom are non-ambulant (GMFCS IV and V) to reduce pain and improve ease of care. A small double-blinded, placebo controlled randomized trial examined the use of Botox^®^ pre-operatively for pain management post hip adductor release surgery for children with severe CP [20]. Compared with the placebo group, children who received Botox^®^ required significantly less pain relief, including morphine (p < 0.03) and combined narcotic dose (p < 0.009) after the first 48 hours post operatively. The study concluded that there is a role for BoNT-A in reducing pain post-surgically and that it may have further clinical applications with this population. A more recent report detailed the outcomes of a clinical cohort of 26 children with CP, GMFCS V with spasticity and pain in the hip region who had injections of BoNT-A (n = 17 had Dysport^®^, n = 9 had Botox^®^) to improve comfort [21]. Pain was measured pre and post treatment by parent report using the Paediatric Pain Profile [22]. All children in the study had reduced pain at 3 months post BoNT-A, with a significant overall reduction in pain (p < 0.001). Although not formally measured, improved sleep patterns, tolerance of seating and ease of care were reported. Another small pre post study of children with severe CP evaluated the effects of intramuscular BoNT-A injections on parent-proxy ratings of pain [23]. The study did not report GMFCS levels of the participants; however 13 of the 34 children had non-ambulant (quadriplegic) CP. Twenty-one (62%) participants experienced pain reduction one month after BoNT-A injections, as measured by a telephone survey with each child’s parent. There were no obvious child-specific characteristics that separated those who had pain reduction and those who experienced persistent pain however this report suggested that BoNT-A injections may have analgesic effects.

The pain-relieving potential of BoNT-A has been more rigorously investigated in adults [24,25]. In a randomized, double blinded placebo controlled trial, BoNT-A injections have been shown to improve shoulder pain associated with spasticity in adults following stroke [24]. Similar results were reported following injections to the pectoralis major muscle in an adult population suggesting that upper limb injections of BoNT-A may provide pain relief [25]. Further well designed studies are needed to validate the efficacy of BoNT-A when used to reduce pain, improve comfort and reduce burden of cares in children with non-ambulant CP.

Following reports of systemic adverse events, [26] there has been increasing focus on the safety of BoNT-A when used with children with CP. A systematic review which included 20 randomized clinical trials of BoNT-A found a good safety profile when used short term, however called for more research into a possible association between BoNT-A, seizures and death [27]. A review of the efficacy and safety of pharmacological treatments for spasticity in children and adolescents with CP reported that BoNT-A was an effective and generally safe treatment however warned that generalized weakness may occur [28]. A retrospective review of 1,980 BoNT-A injection episodes in 1,147 children ranging from GMFCS I –V found a low incidence of systemic adverse events [29]. Incontinence occurred in 1% of cases, and respiratory symptoms requiring hospitalization in 1.3%. Higher GMFCS levels (IV,V) and higher doses of BoNT-A were associated with increased incidence of adverse events, leading the authors to recommend conservative dosing for children classified GMFCS IV-V [29].

A meta-analysis of the safety of BoNT-A using a variety of preparations included 37 randomized controlled trials across a range of indications including participants with dystonia, movement disorders, spasticity and CP, urological and gastrointestinal disorders and for cosmetic use [30]. Data from 2,361 subjects of whom 1,447 had received BoNT-A were reviewed. The incidence of side effects was 25% in BoNT-A treated patients and 15% in the control group. Of the side-effects, only focal muscle weakness and ptosis occurred more frequently in the treatment group [30]. Another retrospective review of 929 patient encounters from a large movement disorders centre reviewed the safety profile of high dose (15-25 units Botox^®^ per kilogram per episode) BoNT-A across domains such as the aetiology of CP, motor pattern, ambulatory potential and muscles injected [31]. The review found adverse effects were randomly distributed across the range of phenotypes and doses. A single blind randomized controlled trial assigned 90 children with bilateral spastic CP, primarily GMFCS IV and V, to either three years of six monthly injections of BoNT-A (maximum dose Botox^®^ 16 units/kg/body weight) combined with wearing a hip abduction brace for 6 hours/day, compared to standard therapy treatment (no brace or BoNT-A). In this study, two children in the treatment group died due to reasons thought not to be related to the BoNT-A injections [32]. Including these two deaths, there were 12 serious adverse events out of 204 injection episodes (6%), including four episodes of respiratory infection, three episodes of transient urinary incontinence, two cases of bronchospasm during recovery from general anaesthesia and one flu-like episode.

Data from these studies suggest that, when used in trial conditions, BoNT-A is a relatively safe treatment for children with severe CP but a small proportion of children may be susceptible to adverse events.

Children with severe CP are at risk of significant comorbidities. O’Flaherty and colleagues conducted an audit of health status in children with CP prior to BoNT-A injections [33]. The incidence of health issues was reported according to GMFCS levels. In the month prior to injections they reported an incidence of 64/178 (36%) of health related issues including respiratory infections and seizures in children classified GMFCS V who were booked for BoNT-A injections (including those whose injections were cancelled for any reason). Liptak and colleagues performed a multicentre study investigating the health status of children with GMFCS V and found those who used a feeding tube had the lowest mental age, required the most health care resources and medications, had the most respiratory problems and the lowest global health scores [34]. The heterogeneity of clinical phenotypes in CP needs to be considered in assessing the efficacy and safety of BoNT-A.

Previous studies of BoNT-A with children with CP have largely focused on children classified GMFCS I – III [13-15,35-43]. In these studies, assessments of motor function have been used to test the ability of BoNT-A to bring about functional improvements. In this study with children with CP who have limited voluntary movement, improvements in motor function are not widely expected and are not the focus of investigation. Children with CP whom are non-ambulant rely on their parents for performance of daily activities. Spasticity and contracture, with associated discomfort and pain, contribute to these tasks being difficult, time consuming and/or stressful for parents. In our clinical BoNT-A injecting program, parents of children with severe CP regularly set goals for intervention around improving ease of care (for example, parents may hope for it to be easier to get their child’s arm through a sleeve, quicker to apply lower limb orthoses, or easier to perform transfers), and improving their child’s comfort (for example, that their child will tolerate sitting in their wheelchair for longer periods, require less pain medication or have fewer night wakings due to discomfort). Efficacy will be determined through measurement of changes in parental perceptions of the ease of carrying out daily cares for their child, and their child’s comfort, pre and post treatment, using the Canadian Occupational Performance Measure (COPM) [44].

Clinically, we observe that reduction of spasticity, improved comfort and easier performance of daily cares has the potential to improve a child’s quality of life, health status and pain. Secondary outcome measures will investigate the impact of injections of BoNT-A and therapy on these factors.

In clinical practice, children who undergo BoNT-A injections typically have repeat injections once the effects have worn off. The majority of studies of BoNT-A with children with CP have investigated a single injection episode however several have examined the efficacy of repeated injections. A study of 22 young children with hemiplegia compared three 16 week cycles of BoNT-A injections followed by occupational therapy, with occupational therapy alone. They found that the group who had BoNT-A had reduced spasticity and improvements in the performance scale of the COPM, compared to the therapy only group. [35]. Another study of young children with hemiplegia included 42 children who had either two or three cycles of upper limb BoNT-A injections and occupational therapy over a 30 month period [14]. First and second injections demonstrated significant effect sizes for quality of movement, goal attainment and functional skills improvement. The study concluded that repeated upper limb injections of BoNT-A are safe and effective for children with unilateral CP receiving occupational therapy. A study of lower limb BoNT-A included ambulant children with CP who were separated into three groups: BoNT-A alone, BoNT-A + casting and casting + placebo injection [45]. Each group received three treatment cycles, with the study finding that the groups who received BoNT-A + casting and casting + placebo obtained successive improvement in ankle kinematics, spasticity, range of motion and strength.

To date, no study has evaluated the efficacy and safety of repeated injections of BoNT-A for children with non-ambulant CP. Cycle II of this study will address this gap through a second phase of treatments, in which all children will receive BoNT-A injections followed by a standard therapy regime, as per cycle I. Outcome measurement and monitoring of safety outcomes will be performed as per cycle I. This will enable testing of the efficacy of repeat injecting and the ability to determine the safety of two episodes compared to one episode of injecting.

Intramuscular BoNT-A is widely used clinically for children with CP whom are non-ambulant [46,47]. Research is urgently needed in order to establish efficacy of intramuscular BoNT-A in meeting child and family goals for improving quality of life by reducing pain and improving care and comfort. This study aims to evaluate the efficacy of BoNT-A, while closely monitoring safety in children with CP whom are non-ambulant.

## Methods/design

### Study design

A double blind randomized controlled trial with two cycles will be conducted to evaluate the efficacy of intramuscular injections of BoNT-A into selected upper and/or lower limb muscles, in 40 children with non-ambulant CP aged 2 to 16 years.

The specific hypotheses to be tested are:-

Cycle I

1. Intramuscular injections of BoNT-A combined with a regime of standard therapy will result in improved parental ratings of performance and satisfaction in areas of concern for their child’s comfort and ease of care, as measured by the COPM, compared to standard therapy alone.

2. Intramuscular injections of BoNT-A combined with a regime of standard therapy will result in greater reduction in pain compared to standard therapy alone.

3. Intramuscular injections of BoNT-A combined with a regime of standard therapy will result in greater improvements in quality of life compared to standard therapy alone.

4. Intramuscular injections of BoNT-A combined with a regime of standard therapy will not result in an increased likelihood of adverse events compared to standard therapy alone.

Cycle II

1. Repeated BoNT-A injections will result in greater overall improvements in pain, quality of life, burden of care and individual family concerns for ease of care and comfort, compared with a single episode of BoNT-A.

2. Repeated BoNT-A injections will not increase the likelihood of an adverse event in a population of children with non-ambulant CP, compared with a single episode of injections.

**Figure 1:**
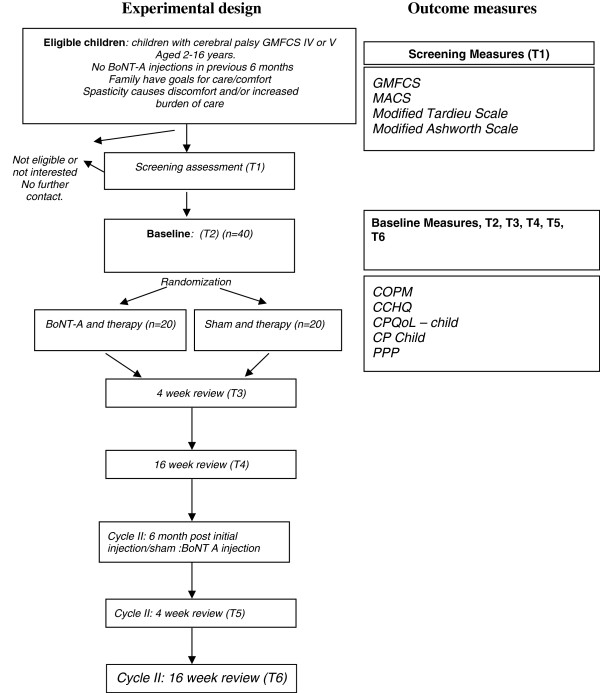
**Flow chart of care and comfort study according to CONSORT guidelines.** Key:- BoNT-A, Botulinum Toxin A. GMFCS, Gross Motor Function Classification System. MACS, Manual Ability Classification System. T1-T6. Time-points 1-6. COPM, Canadian Occupational Performance Measure. CPQOL-Child, CP Quality of Life – Child. CCHQ – Child Health Questionnaire. PPP – Paediatric Pain Profile.

Assessments will be performed at baseline, 4 weeks and 16 weeks following injections/sham in cycle I and at 4 weeks and 16 weeks following injections in cycle II. There will be two months between cycles, resulting in a total study duration of ten months. The experimental design and outcome measures are depicted in Figure 1.

The research ethics board for the Queensland Children’s Health Services, at the Royal Children’s Hospital, Brisbane (EC 00175) has granted approval for the study.

### Study sample and recruitment

Children and youth will be recruited from across Queensland, Australia. Financial support for families from regional areas will be made available to allow equity of access to the program and a representative sample of children from metropolitan, outer metropolitan and rural/regional/remote areas. Children will be identified as potential candidates for the study by their treating Paediatrician or Rehabilitation Specialist at routine attendance at multidisciplinary clinics. A screening assessment will be performed by the child’s treating Physician to determine eligibility.

### Inclusion criteria

The study will include children and youth:

1. With a confirmed diagnosis of CP classified functionally as GMFCS level IV or V;

2. Aged 2-16 years at study entry;

3. With goals primarily concerned with improving ease of care and/or comfort;

4. With spasticity in the upper and/or lower limbs causing discomfort and/or increased burden of care.

### Exclusion criteria

1. Predominant ataxic or hypotonic motor type;

2. Previous severe adverse event following BoNT-A injections;

3. Medical contraindications to BoNT-A such as history of allergic reaction to BoNT-A;

4. BoNT-A injections or orthopaedic surgery occurring within 6 months prior to commencement of the study;

5. If changes to oral or other antispasticity treatment (e.g. intrathecal baclofen, oral baclofen) occur within two months of the commencement of the study, entry will be delayed until treatment has been stable for two months.

### Sample size

Sample size calculations were primarily based on the findings from a placebo controlled trial of the analgesic effects of BoNT-A in a sample of 16 children with marked CP [20]. In this study, the response within each subject group was normally distributed with a standard deviation of 0.56. The mean difference in pain scores between the experimental and control groups at follow-up was 0.74. If the true difference in experimental and control means is 0.74, we will be able to reject the null hypothesis that the population means of both groups are equal with probability (power) .981. The type 1 error associated with this test of this null hypothesis is 0.05. We also examined a study of upper limb BoNT-A injections and occupational therapy, with a heterogeneous sample of 72 subjects that included 28 (39%) children with quadriplegic (CP) [13]. In this study, COPM outcomes at 3 months post injection resulted in a SD = 1.6. A difference of 2 points on the performance and satisfaction scales of the COPM, 80% power and significance level of 0.05 gives a sample size of 24 (12 in each group). Based on these previous studies and factoring in a buffer for drop-outs, we plan to recruit 20 participants to each group (total sample 40 subjects).

For the secondary hypothesis of safety in our double blind sham controlled trial with 20 subjects in each group, we examined a recent single blind randomized controlled trial of BoNT-A in children with bilateral CP [32]. That study reported a 6 percent rate of serious adverse events in the BoNT-A group (failure rate in controls of 0.01). If the true failure rate in the experimental group is 0.333 then we will be able to reject the null hypothesis that the failure rates for experimental and control subjects are equal in probability (power) 0.797 with a type I error of 5 percent using an uncorrected chi-squared statistic.

### Randomization

Children will be randomly allocated to the treatment or control group. Randomization will be performed using concealed allocation. Prior to randomization, participants will be stratified according to primary goal areas (upper limb or lower limb) in order to allow block randomization with the intention that similar numbers of children with predominantly upper and lower limbs injected will be in each arm of the study.

### Study treatments

Muscles for injection with BoNT-A will be selected on the basis of the parent’s/carer’s priorities for improving ease of care and comfort, as well as musculoskeletal examination of range of motion and spasticity, using the Modified Tardieu Scale [48] and the Modified Ashworth Scale [49]. The BoNT-A dosing regime was determined with reference to the European consensus table for the use of BoNT-A with children with CP [50], which suggests a safe range of 6-25 units/kg/body weight with a total dose of 400-600 units of Botox^®^. These recommendations were modified slightly when reviewed in 2009 [51] to 1-20 units/kg/body weight, suggested maximum dose 400 units of Botox^®^, with caution urged when planning doses for children with significant co-morbidities. Injections will be guided by localization with ultrasound [52] and/or muscle stimulation [53]. Doses will be 0.5-4 units/kg/muscle group as clinically indicated to maximum dose of 12 units/kg/ body weight (or 400 units maximum) of Botox^®^. A sham procedure was chosen for the control group rather than placebo injections, so as to minimize pain and discomfort for the participants. All injections and sham procedures will be performed on a day procedure unit with a trained, experienced Physician and two Clinical Nurse Consultants (CNCs) in attendance. An alternative Physician in the investigative team (not the child’s regular Physician) will carry out the procedure, to ensure blinding of the treating Physicians who will carry out the review assessments. The injecting Physician will be assisted by a CNC. A second CNC will monitor the child during the procedure. Parents will read a book to their child while the procedure is being carried out, to provide distraction. A screen blocking the parent’s, child’s and monitoring CNC’s view of the procedure will ensure that they remain unaware of group allocation.

### Sedation/pain management during BoNT-A injections

When children with non-ambulant CP have BoNT-A injections at our centre, a brief general anaesthetic is typically administered. The side-effects of general anaesthesia can be similar to those considered to be possible side effects of BoNT-A, including respiratory complications. A study by Naidu et al (2010) reviewed 1980 episodes of BoNT-A treatment and found that while the incidence of respiratory complications was low, there was a relationship to functional severity (GMFCS level), with increasing GFMCS levels associated with increased likelihood of unplanned hospital admission due to respiratory complications [29]. Most of the children in the review had inhalational anaesthesia, thus the respiratory complications may have resulted from the anaesthetic procedure, leading the authors to suggest that for children with non-ambulant CP, alternatives to mask anaesthesia should be considered.

In order to more accurately ascribe adverse effects to BoNT-A, participants in this study will be given intranasal fentanyl (1.5mcg/kg / 300mcg/ml solution) for pain relief 10 minutes prior to the procedure, as an alternative to general anaesthesia. Fentanyl is a short acting lipophilic opioid analgesic. Previous reports have documented the safety and effectiveness of intranasal fentanyl in paediatric and adult patients for management of painful procedures such as burns dressings, [54,55] management of acute pain such as fractures, [56,57] and postoperative analgesia [58-60]. Local pain relief will be achieved by the application of Eutectic Mixture of Local Anaesthetic (EMLA) to marked sites one hour prior to injections. All injection sites will be covered with adhesive dressings and tincture of iodine to mask the injection site.

### Sham procedure

Children will have EMLA topical anaesthetic cream applied to marked injection sites one hour prior to the sham procedure. The administration of intranasal fentanyl will be mimicked by the administration of an equivalent amount of intranasal saline, 10 minutes prior to the sham procedure. The procedure will be mimicked with ultrasound and a blunt needle (not penetrating the skin) to the sites selected for injection, by a Physician. The purpose of this is to imitate true time and actions of the actual procedure to assist with blinding. All sites will be covered with adhesive dressings and tincture of iodine, to mask the sham injection site.

### Therapy protocols and delivery

There are a lack of clinical BoNT-A trials that provide evidence for optimal adjunctive therapy with children with CP whom are non-ambulant. Consistent with international consensus statement recommendations, all participating children will receive a block of therapy treatment commencing within two weeks following BoNT-A or sham [19,61]. Therapy will be provided by experienced occupational therapists and/or physiotherapists. Treatments will aim to address the concerns and priorities identified by families at the initial screening visit, typically the reduction of impairments and symptom management to ease pain and discomfort. Therapy regimes will be based on international consensus recommendations [19,61,62] and will include practices such as: serial casting to lengthen muscles and reduce contractures, [45,63-66] strengthening of injected muscles and antagonists, [67] provision of stretching programs to be implemented into the child’s daily routines, [68] provision of splints/orthoses, [69] and/or targeted motor training. It is recognized that children with non-ambulant CP will have difficulties participating in movement based therapy. Provision of strengthening activities and targeted motor training in a hydrotherapy medium will be provided in order to enable participation in therapy programs [70]. The therapy plan will be determined *apriori* at the screening clinic, prior to randomization. Treating therapists will be masked to group allocation. Children in both groups will receive the same dose of therapy, consisting of up to two weeks of serial casting if clinically indicated, and four, one hour therapy sessions.

### Monitoring of safety and adverse events

A pre-admission medical check detailing comorbidities and health status will be conducted by the assessing Physician at entry and the injecting Physician. During the procedure, a nurse who is masked to group allocation (VW) will collect a record of in-procedure adverse events and observations. Subsequently, information about adverse events will be collected via a standardized questionnaire at the following time points: immediately following the procedure, 2 weeks following procedure (via phone call), and at the 4 week and 16 week review appointments. All adverse events data will be reported to one of the blinded study investigators (PE), who will grade each event, according to the following grading system which is consistent with the Common Terminology Criteria for adverse events, version 4.0:

1. Mild – awareness of sign or symptom, observations only, intervention not indicated.

2. Moderate – discomfort causing interference with usual activity, minimal or local intervention indicated.

3. Serious/severe – medically significant, requirement for IV therapy, prolongation of procedure or hospitalization requirement within three months of intervention.

4. Sentinel – Death or disability with a temporal relationship to procedure [71].

If a sentinel event occurs monitoring of patients and cessation of program until external review will be mandatory.

Adverse events will be additionally categorized according to a graded probability rating scale which considers the causality/relationship of the event to the interventions in the procedure in addition to the following: comorbidities, temporal relationship, history of medical events and family history. The scale will rate events as definitely/probably, possibly, or unlikely/un-related to the intervention [71]. Serious adverse events with suspected causal relationship to the intervention will be notified to an external clinical pharmacologist and the Ethics Committee within 72 hours. The external assessor/ clinical pharmacologist will review all adverse events every 16 weeks, unmasked to group allocation.

### Outcome measures and procedures

1. Classification of the sample:

The participants entered into the study will be classified according to:

a) Gross Motor Function Classification System (GMFCS)

The GMFCS classifies severity of CP in terms of gross motor function [2]. A child is assigned one of five levels to describe their ability in self-initiated movements, with a focus on sitting and walking. The GMFCS has good construct validity with the Gross Motor Function Measure (r = -0.91) [72] and clinical relevance [73]. Inter-rater reliability is reported to be high [74]. All children in this study will be classified GMFCS IV or V (non-ambulatory).

b) Manual Ability Classification System (MACS)

The MACS is a five-point scale corresponding to the structure of the GMFCS, which classifies how a child uses their hands to perform day-to-day activities that are appropriate for their age [75]. Inter-rater reliability of the MACS is excellent as reported by Eliasson et al., 2006 (ICC = 0.97 (0.96 – 0.98)) [75]. It is expected that most children in the study will be MACS level V (does not handle objects) or IV (handles a limited selection of easily managed objects).

2. Assessment of spasticity and range of motion:

To aid identification of appropriate muscles to be treated with BoNT-A, spasticity in the upper and lower limbs will be assessed with the Modified Tardieu Scale (MTS) [48] and passive resistance of the muscle with the Modified Ashworth Scale (MAS) [49]. Passive range of motion of the limbs will also be recorded. Muscles that are assessed to have spasticity and appear to impact on identified goals will be selected for injection. For example, if extending the arms for dressing is identified as being an area of difficulty for carers and the elbow flexors are determined to be spastic, injections to biceps brachii, brachialis and/or brachioradialis will be planned. If lower limb dressing is hindered by difficulty abducting the legs due to spasticity in the hip adductors and medial hamstrings, injections to these muscle groups will be performed.

3. Primary and Secondary Outcome Measures:

The primary outcome measure for this study is change in parent ratings in identified areas of concerns for their child’s care and comfort, using the Canadian Occupational Performance Measure (COPM). Secondary measures include measures of pain, ease of care, health status and quality of life. To maximise reliability in the study, the same person (the child’s primary caregiver) will be responsible for completing the measures at each assessment point.

a) Ease of Care

The primary outcome to assess ease of care and comfort will be the Canadian Occupational Performance Measure (COPM) [44]. The COPM is a client-centered tool which aims to identify concerns and issues of occupational performance, incorporating self-maintenance (activities of daily living, functional mobility, and community management), productivity (participation at school and in play) and leisure (quiet recreation, active recreation and socialization). The COPM allows for consistent measurement of a broad range of issues unique to children and families that may not be detected by standardized measures. This is important as our sample will be heterogeneous in terms of motor presentation (GMFCS IV and V), degree of spasticity and contracture, and age (2-16 years). The COPM has been used previously in studies of efficacy of intramuscular BoNT-A injections [13,15,35].

The COPM is a semi-structured interview conducted with parents, by a clinician skilled in its use. The modifications for using the COPM with a paediatric sample suggested by Cusick et al [76] will be applied in this study. For each participant, a parent will be asked to report areas of concern for their child’s care and comfort in the domains of self-maintenance, productivity (school/play) and leisure. Parents will then select a minimum of two of the most important areas that impact upon burden of care or discomfort and rate their concerns on two 1-10 ordinal scales (performance and satisfaction). Follow-up assessments require that parents re-rate their concerns according to performance and satisfaction, without reference to their original ratings, so as not to bias their responses [77]. A two point change in total performance or satisfaction has been shown to indicate clinical significance [44]. The COPM has been shown to be valid [44,78,79] and reliable [80] with high test/re-test reliability (Spearman’s Rho 0.89 for performance scores, 0.88 for satisfaction scores) [81]. The COPM takes approximately 20 minutes to complete.

In addition to parent report of unique individual concerns, ease of care and comfort will be assessed via the Care and Comfort Hypertonicity Questionnaire (CCHQ) [82]. The CCHQ uses a seven point scale and will be used to measure specific aspects of ease of care and comfort in the areas of personal care, positioning/transferring, comfort and interaction/communication. The CCHQ has been designed for use with children with severe disabilities associated with hypertonicity of cerebral origin. Content validity has been established and responsiveness to change has been shown for intrathecal baclofen treatment with children with severe CP [82]. It takes approximately 10 minutes to complete.

b) Quality of life

Quality of life is a broad term encompassing well-being across a variety of domains including physical and psychological status, function, social interactions and economic status [83]. Quality of life is important to measure in clinical studies with children with CP, as the relationship between clinical indicators of disability and the subjective experience of disability is not clear [83].

We will measure quality of life pre and post intervention with the Children with Cerebral Palsy Quality of Life Questionnaire (CP QoL–Child) [84]. The CP QoL-Child is a condition specific quality of life tool designed for use for children with CP aged 4-12 years. Both a parent proxy version and a child self- report version are available. The parent version will be used for this study. Domains include: Social Wellbeing and Acceptance; Feelings about Functioning; Participation and Physical Health; Emotional Wellbeing, Access to Services; Pain and Impact of Disability and Family Health. Internal consistency ranges from 0.74-0.92 [85]. Test re-test reliability ranges from 0.76-0.89 [85]. The CP QoL-Child takes approximately 10-15 minutes to complete. A recent systematic review of CP-specific quality of life measures concluded that the CP QoL-Child had the strongest foundation in the theoretical basis of quality of life, measuring quality of life across broad domains [86].

c) Health status

Children with non-ambulant CP are at risk of multiple health related problems [34] which lead to activity restrictions. In this study, health status will be measured using the Caregiver Priorities and Child Health Index of Life with Disabilities (CPCHILD^©^) Questionnaire [87], which was developed to measure health status and wellbeing of children with severe CP, from the perspective of caregivers. The tool consists of 37 items over 6 domains: Personal Care; Positioning, Transferring and Mobility; Comfort and Emotions; Communication and Social Interaction Health; & Overall Quality of Life. Ordinal scales are used within each domain. Developed as an evaluative tool, initial reports of its psychometric properties suggest that it is a valid measure of parent’s perceptions of their child’s health status, functional limitations and well-being, and test re-test reliability of the total questionnaire score was high (ICC 0.97 (0.95-0.99)) [87]. To date, the CPCHILD has not been used in BoNT-A outcome studies. It takes approximately 15 minutes to complete.

d) Pain

Injections of BoNT-A may reduce pain in children with non-ambulant CP [20]. We will assess pain using the Paediatric Pain Profile (PPP) [22]. The PPP is a 20 item behaviour rating scale designed to assess pain in children with non-ambulant CP and other severe neurological impairments. Behaviours include changes in facial expressions, vocal sounds, posture and movements, daily routines and mood. The tool has established validity for use with children with neurological conditions [88]. Intra-rater reliability for total scores (ICC = 0.90) is reported to be better than inter-rater reliability (ICC = 0.62 - 0.83) [88].

### Analyses

#### Cycle I- efficacy of BoNT-A injections for improving ease of care and comfort

Analysis will follow standard principles for RCTs, using two-group comparisons on all participants on an intention to treat basis. Data from each outcome measure will be summarized for each group and descriptive statistics (frequencies, means, medians, 95% confidence intervals) calculated dependent on data distribution. We anticipate that groups will be similar on baseline measures. The primary comparison for hypothesis one at 4 weeks will be the COPM performance and satisfaction scores. Generalised estimating equations (GEEs) will be used to compare treatment groups at follow-up, with time (0, 4 and 16 weeks) and study group, as well as a time by group interaction as covariables [89]. We will use the Gaussian family, identity link, and an unstructured correlation structure. Secondary analyses will compare the outcomes between groups for ease of care, quality of life, pain and health status using GEEs as outlined above (STATA 11).

#### Cycle I: safety of BoNT-A injections compared with sham

Two Chi-squared tests of independence will be conducted to assess the relationship between treatment and sham and all adverse events vs. moderate and serious events. The continuity correction chi-square will enable analysis by 2 × 2 tables using Fisher’s exact test.

#### Cycle II: efficacy of repeated episodes of BoNT-A injections vs. single episode

The primary outcome measure for cycle II will be the COPM administered at 4 weeks post injections. The secondary outcome measures will be the same as cycle I (CPCHILD, CP QoL-Child, CCHQ and PPP). Generalised estimating equations (GEEs) will be used to compare treatment groups at follow-up, with time (0, 16 weeks and 10 months) and study group, as well as a time by group interaction as covariables. We will use the Gaussian family, identity link, and an unstructured correlation structure. Secondary analyses will compare the outcomes between groups for ease of care, quality of life, pain and health status using GEEs as outlined above (STATA 11).

#### ***Cycle II: safety of repeated episodes of BoNT-A injections vs. single episode***

Adverse events in cycle II will be collected as per cycle I. Rates of adverse events will compared between groups using Poisson regression.

## Discussion

This paper presents the background and design for a double blind, randomized controlled trial. This study is the first to rigorously examine the efficacy and safety of BoNT-A in addition to standard therapy to improve care and comfort for children with non-ambulant CP using a strong study design to minimize bias. Outcomes will be measured using valid and reliable measurement tools and the double blind sham study design will further strengthen the attribution of effects and monitoring of safety.

## Competing interests

The authors declare no conflicts of interest.

## Authors’ contributions

MT, PE, LC, MK, JL, LZ and RB contributed to study concept and design. MT and RB drafted the manuscript. The manuscript was critically reviewed by all authors. SD, LG, LC, PE, KM, JL and MK contributed to acquisition of data. MK obtained funding for the study. Administration, technical and material support was carried out by SD and LG. RB supervised the study. All authors read and approved the final manuscript.

## Pre-publication history

The pre-publication history for this paper can be accessed here:

http://www.biomedcentral.com/1471-2431/12/120/prepub
